# Supplementary material for: Structural and Evolutionary Analysis Indicate That the SARS-CoV-2 Mpro Is a Challenging Target for Small-Molecule Inhibitor Design
Source: Int J Mol Sci. 2020 Apr 28;21(9):3099. doi: 10.3390/ijms21093099 (PMC7247150; doi:10.3390/ijms21093099)
Supplement: Supplementary file 1 [file ijms-21-03099-s001.pdf]

SUPPLEMENTARY INFORMATION

Structural and Evolutionary Analysis Indicate that the SARS-CoV-2 Mpro is a Challenging Target for Small-Molecule Inhibitors Design

Maria Bzówka[1#], Karolina Mitusińska[1#], Agata Raczyńska[1], Aleksandra Samol[1], Jack A. Tuszyński[2,3], Artur Góra[1*]

1) Tunneling Group, Biotechnology Centre, ul. Krzywoustego 8, Silesian University of Technology, Gliwice, 44-100, Poland
2) Department of Physics, University of Alberta, Edmonton, AB, T6G 2E1, Canada
3) DIMEAS, Politecnico di Torino, Corso Duca degli Abruzzi, 24, Turin, 10129, Italy

*Corresponding author: a.gora@tunnelinggroup.pl
[#]These authors contributed equally to this work

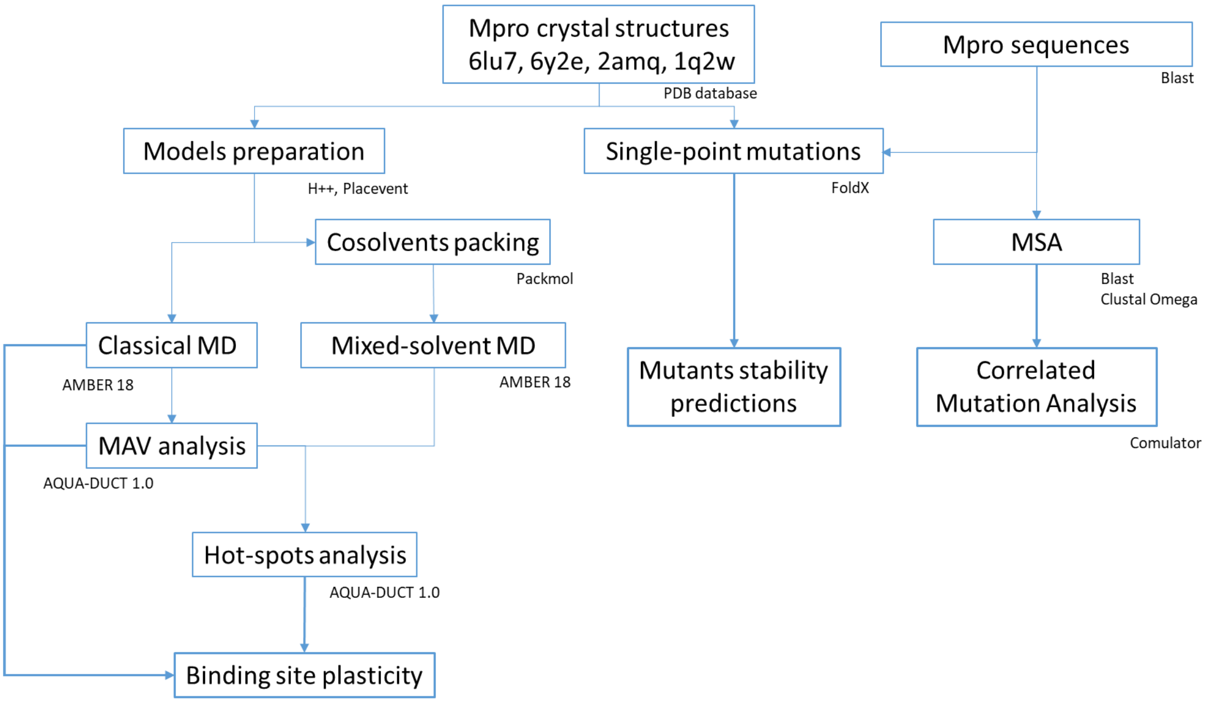

Supplementary Figure S1. The experiment setup and methodology workflow.

Supplementary Table S1. Differences between SARS-CoV-2 and SARS-CoV Mpros proteins. The last column shows differences in total energies (in kcal/mol) calculated as differences in Gibbs free energy folding between SARS-CoV Mpro and introduced single-point mutations as they are in SARS-CoV-2 Mpro structure.

ID	SARS-CoV-2 Mpro	SARS-CoV Mpro	domain	buried/exposed (based on the NetSurfP calculations)	total energy differences [kcal/mol] (based on the FoldX calculations)
35	V	T	I	B	0.90
46	S	A	I	B	0.14
65	N	S	I	B(SARS-CoV-2)/ E(SARS-CoV)	0.32
86	V	L	I	B	2.76
88	K	R	I	E	-0.42
94	A	S	I	E	-0.41
134	F	H	II	E	-0.85
180	N	K	II	E	1.29
202	V	L	III	B	1.78
267	S	A	III	B	2.18
285	A	T	III	E	-0.37
286	L	I	III	E	-0.08
Changes in the protein's sequence, not present in the crystallographic structure					
305	F	Q		E	
306	Q	G		E	

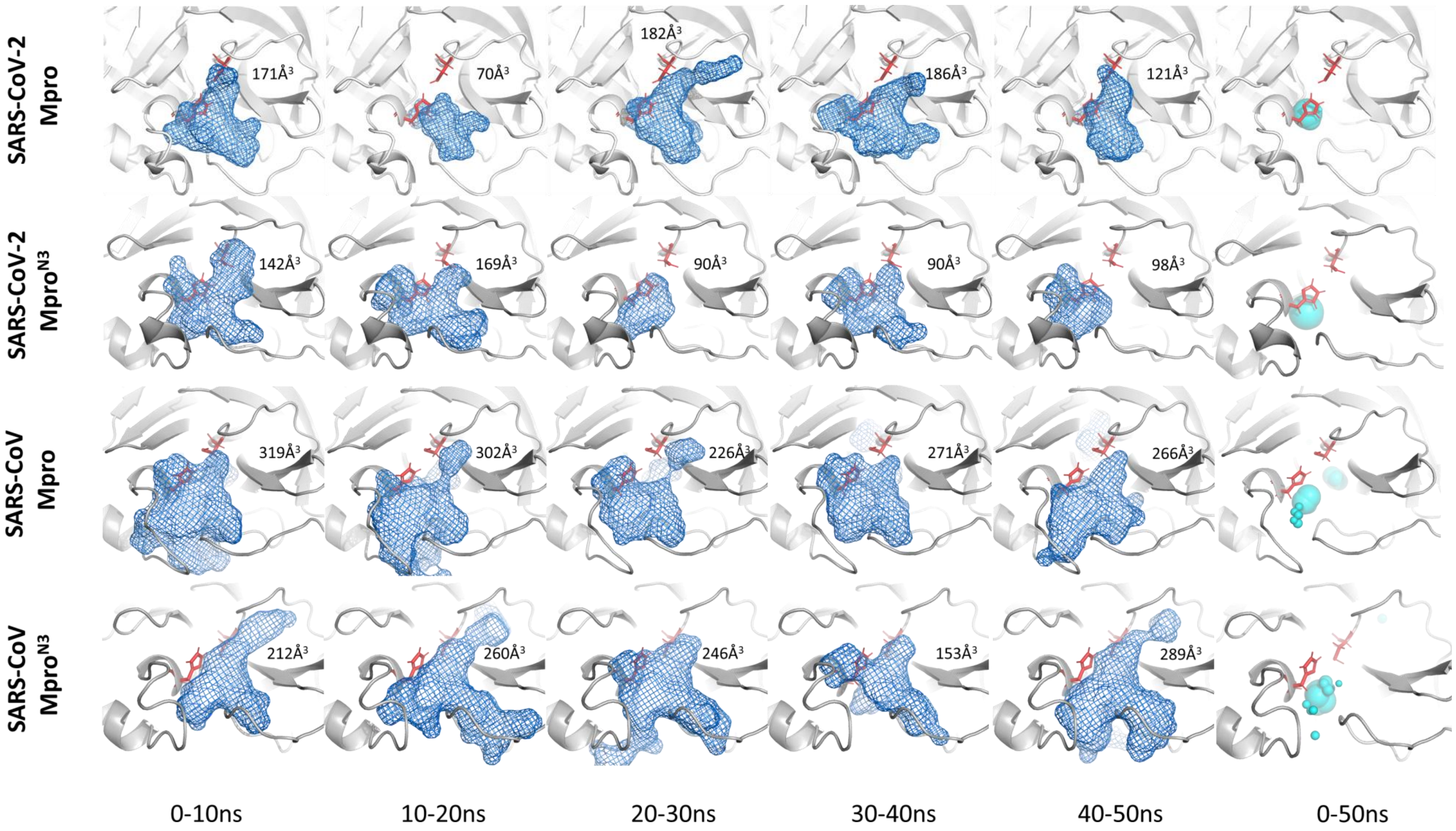

Supplementary Figure S2. The example of time mode analysis of the maximal accessible volume (MAV) (blue mesh) of Mpro structures. The catalytic dyad is shown as red sticks. The last column shows the average location of water hot-spots (cyan spheres) during the simulation time. The position of the biggest hot-spot in each row reflects the position of the catalytic water molecule.

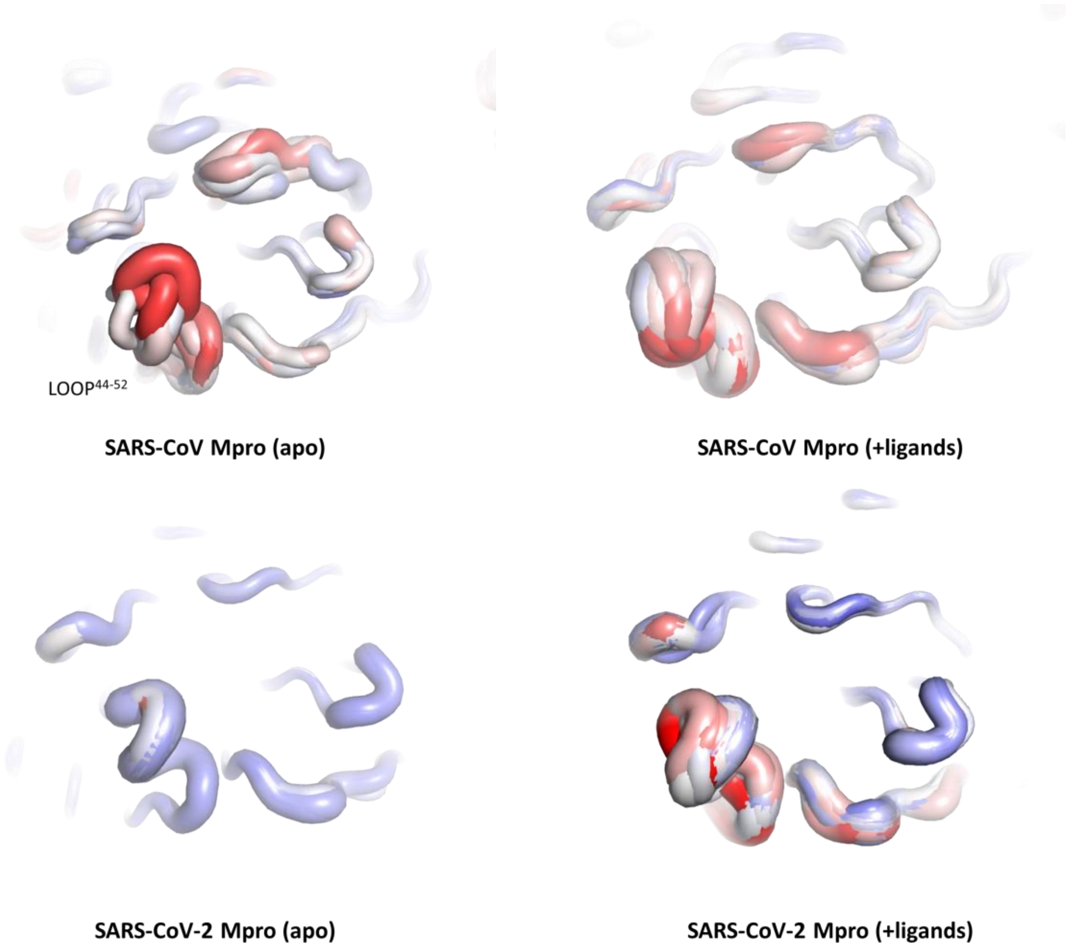

Supplementary Figure S3. Flexibility of loops surrounding the entrance to the binding cavity of according to b-factor assigned to crystal structures deposited in PDB database: SARS-CoV apo: 1q2w, 1uj1, 1uk2, 1uk3, 1z1i, 2a5a, 2bx3, 2bx4, 2c3s, 2duc, 2gt7, 2gt8, 2gz9 and 2h2z, SARS-CoV with inhibitors: 1uk4, 1wof, 2a5i, 2a5k, 2alv, 2amd, 2amq, 2d2d, 2gtb, 2gx4, 2gz7, 2gz8, 2hob, 2op9, 2v6n, 2vj1, 2z3c, 2z3d, 2z3e, 2zu4, 2zu5, 3avz, 3d62, 3sn8, 3sna, 3snb, 3snc, 3snd, 3sne, 3szn, 3tit, 3tiu, 3tns, 3tnt, 3v3m, 3vb3, 3vb4, 3vb5, 3vb6, 3vb7, 4mds, 5n19, 5n5o, SARS-CoV-2 apo: 5r8t, 6m03, 6m2q, 6y2e, 6y64 and 6yb7, SARS-CoV-2 with inhibitors: 5fr0, 5r7y, 5r7z, 5r80, 5r81, 5r82, 5r83, 5r84, 5re4, 5re5, 5re6, 5re7, 5re8, 5re9, 5rea, 5reb, 5rec, 5red, 5ree, 5ref, 5reg, 5reh, 5rei, 5rej, 5rek, 5rel, 5rem, 5ren, 5reo, 5rep, 5rer, 5res, 5ret, 5reu, 5rev, 5rew, 5rex, 5rey, 5rez, 5rf1, 5rf2, 5rf3, 5rf4, 5rf5, 5rf6, 5rf7, 5rf8, 5rf9, 5rfa, 5rfb, 5rfc, 5rfd, 5rfe, 5rff, 5rfg, 5rfh, 5rfi, 5rfj, 5rfk, 5rfl, 5rfm, 5rfn, 5rfo, 5rfp, 5rfq, 5rfr, 5rfs, 5rft, 5rfu, 5rfv, 5rfw, 5rfx, 5rfy, 5rfz, 5rg0, 5rg1, 5rg2, 5rg3, 5rgg, 5rgh, 5rgi, 5rgj, 5rgk, 5rgl, 5rgm, 5rgn, 5rgo, 5rgp, 5rgq, 5rgr, 5rgs, 6lu7, 6m2n, 6w63, 6y2f, 6y2g and 6y7m Please take into consideration, that the quality of particular crystal structures can differ substantially.

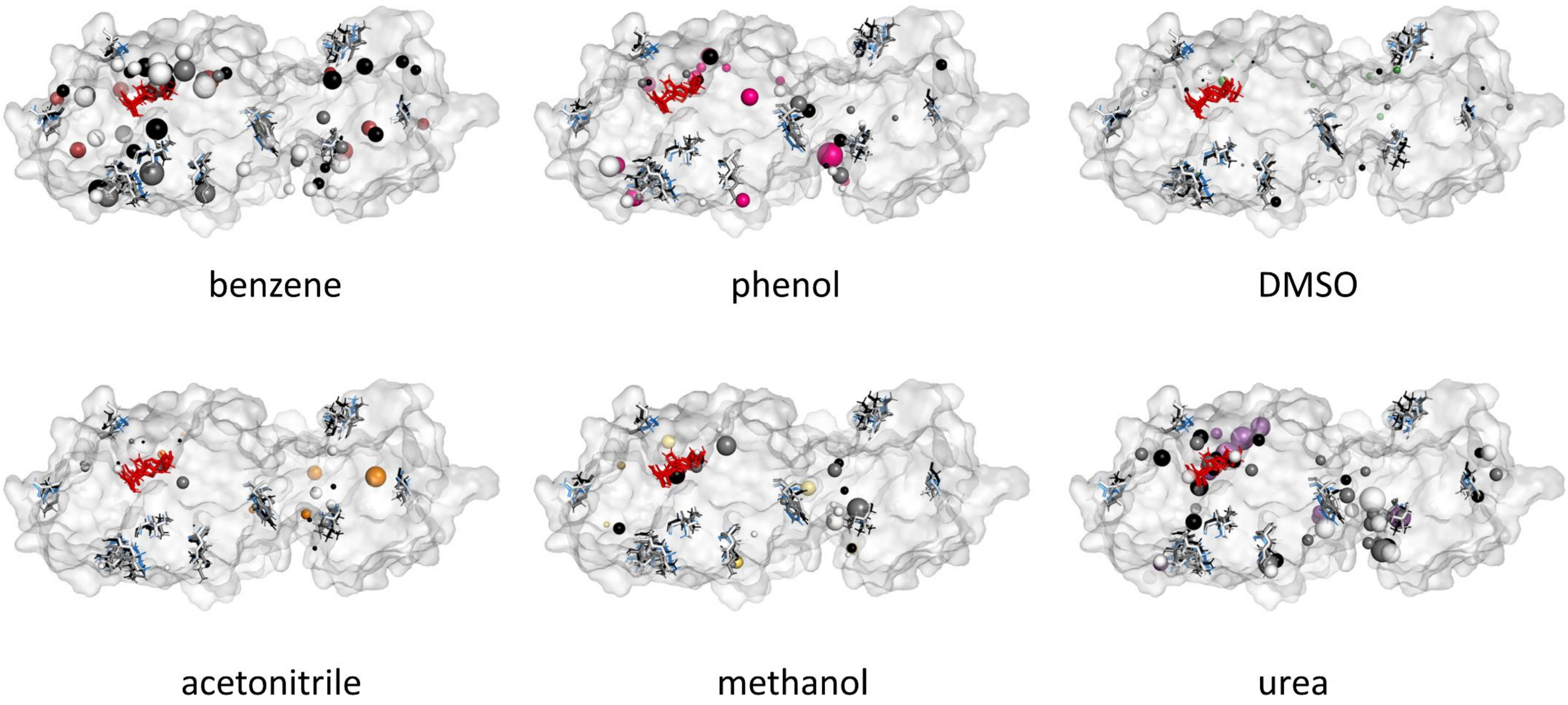

Supplementary Figure S4. Localisation of the global hot-spots of all analysed Mpros. SARS-CoV Mpro^{N3} and SARS-CoV Mpro. The structures of all analysed Mpro structures are superposed and the colour-coding is as follows: orange, red, yellow, green, pink and purple hot-spots are from the SARS-CoV-2 Mpro^{N3}, white hot-spots from the SARS-CoV-2 Mpro, black hot-spots from the SARS-CoV Mpro^{N3}, and grey hot-spots from the SARS-CoV Mpro structure. The active site residues are shown as red sticks, the differing residues of the SARS-CoV-2 Mpro as blue sticks, and the proteins' structures are shown in surface representation.

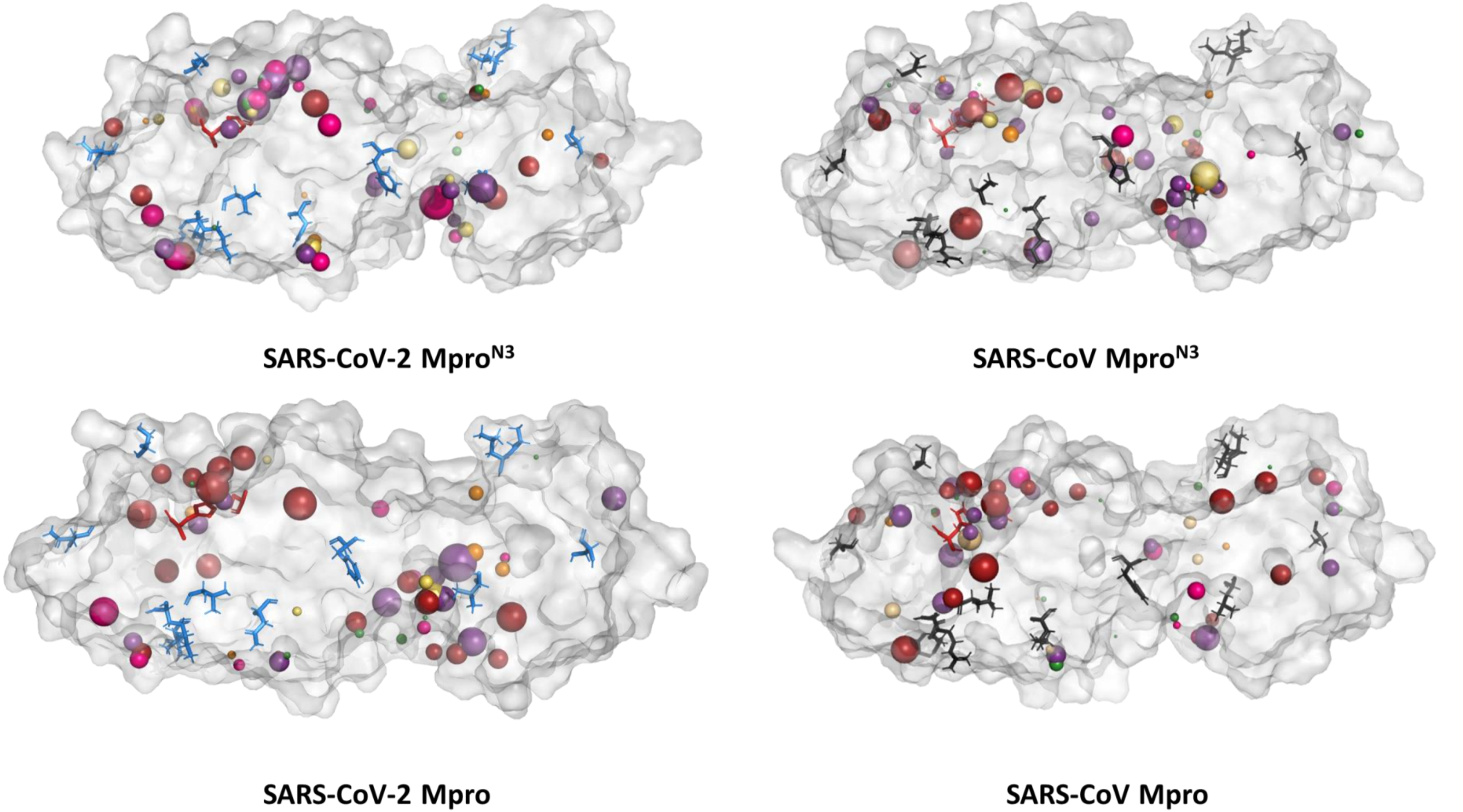

Supplementary Figure S5. Localisation of the global hot-spots identified in the binding site cavities in SARS-CoV-2 and SARS-CoV main proteases. Hot-spots of individual cosolvents are represented by spheres, and their size reflects the hot-spots density. The colour coding is as follows: purple - urea, green - dimethylsulfoxide, yellow - methanol, orange - acetonitrile, pink - phenol, red - benzene.

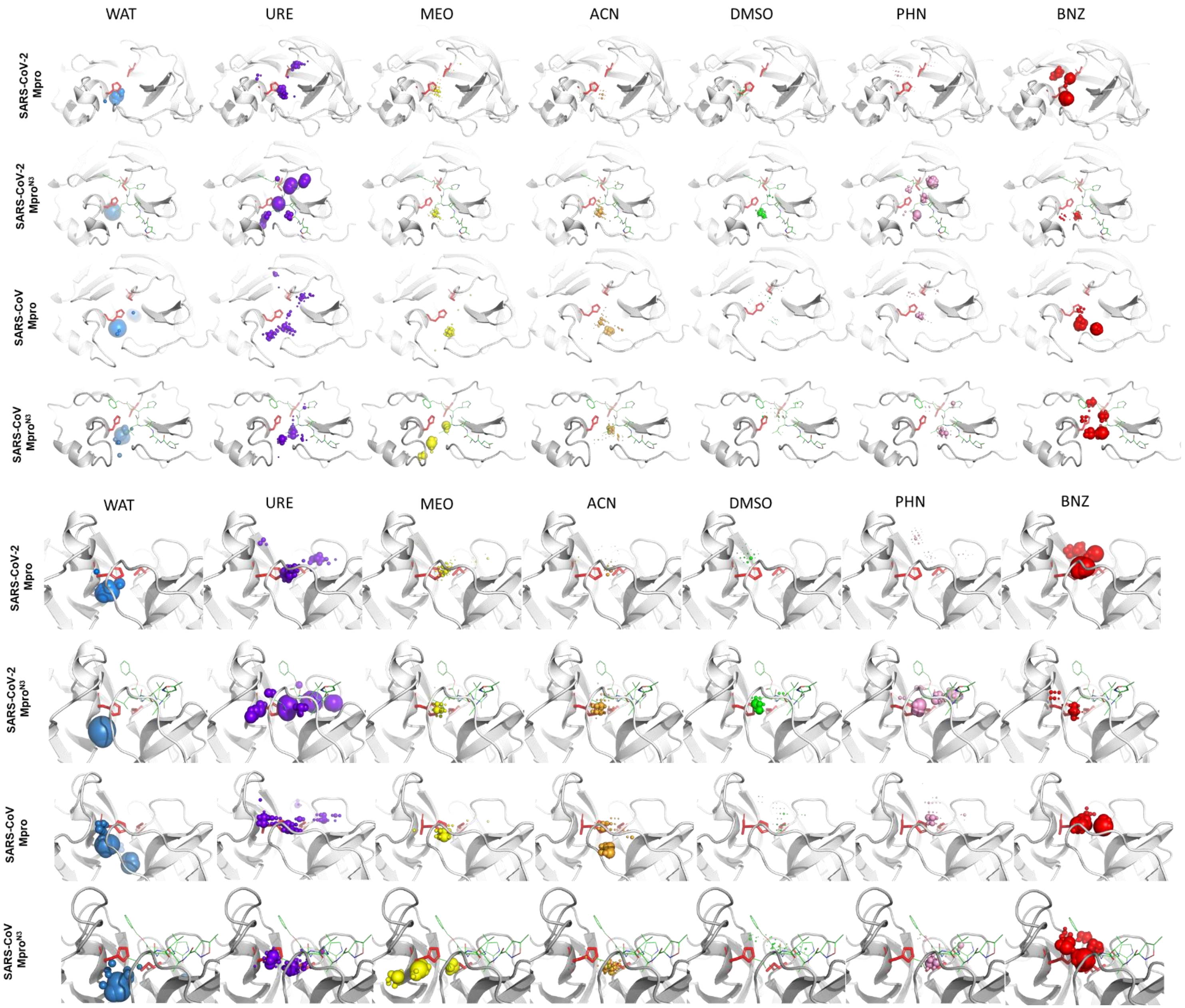

Supplementary Figure S6. Localisation of the local hot-spots of all analysed Mpros: COVID-19 Mpro, SARS-CoV Mpro and SARS-CoV Mpro-f. Hot-spots for individual cosolvents are represented by spheres, and their size reflects the hot-spots density. The colour-coding is as follows: purple - urea, green - DMSO, yellow - methanol, orange - acetonitrile, pink - phenol, red - benzene. The active site residues are shown as red sticks, the N3 inhibitor structure as green lines, and the proteins' structures are shown in cartoons representation.

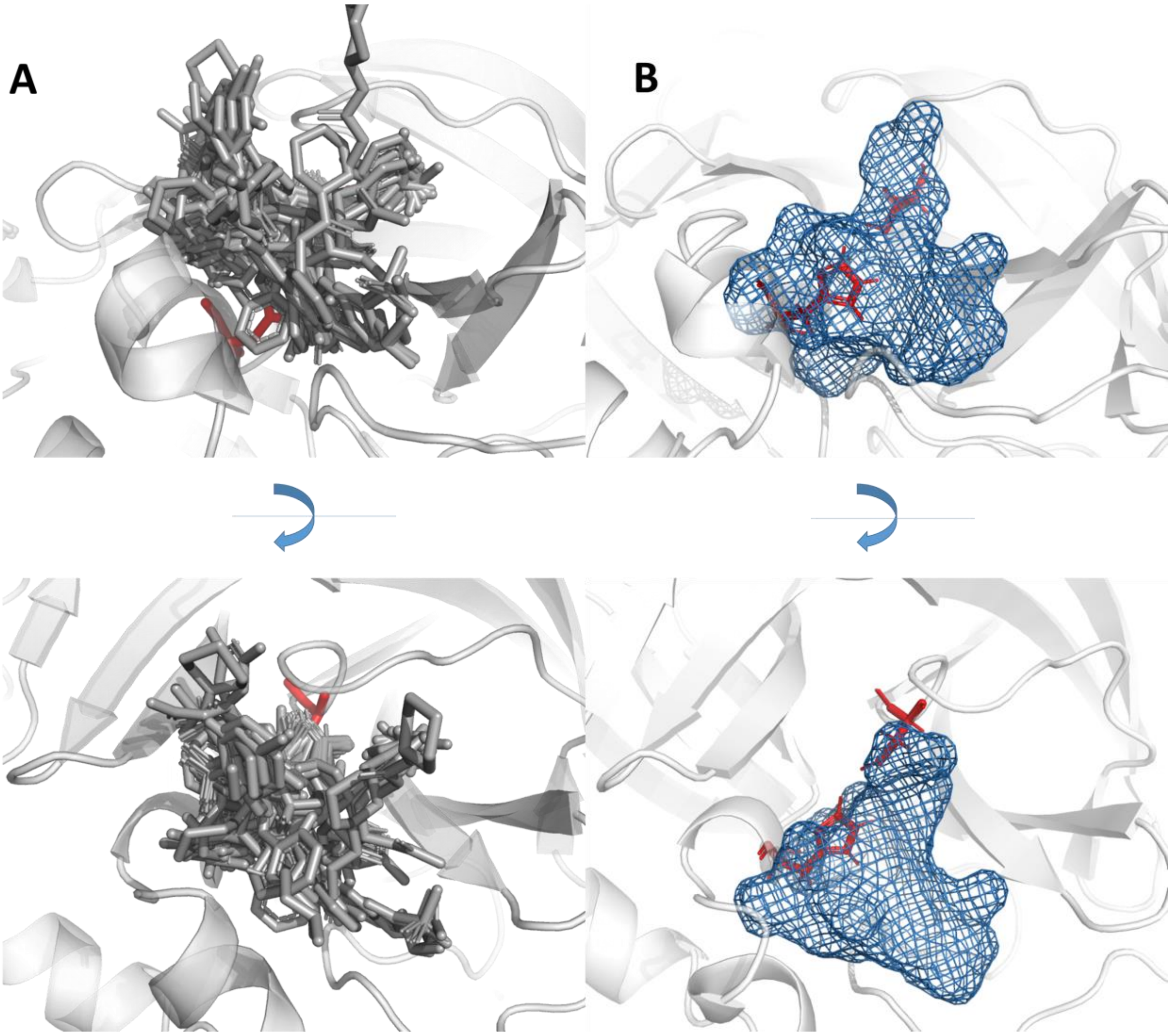

Supplementary Figure S7. Comparison of the space occupied by covalently bound fragments in the active site cavity (Diamond Light Source group) (A) with maximal accessible volume calculated by AQUA-DUCT software (B). It is noted, that part of the ligands extends beyond the protein surface.

Supplementary Table S2. FoldX results for the most energetically favourable potential mutations in the SARS-CoV-2 Mpro structure. Amino acids from the binding cavity are marked **bold**.

Mutation	Energy difference [kcal/mol]	buried/exposed*
A260D	-3.67	E
Y154H	-2.04	E
T21I	-2.01	B
**H41L**	**-1.95**	**B**
Q127L	-1.89	E
A194P	-1.82	E
A129V	-1.75	B
Q306R	-1.73	E
**H164L**	**-1.71**	**B**
S301L	-1.68	E
V233L	-1.58	B
Q244P	-1.54	E
N53D	-1.51	E

* based on the NetSurfP calculations

Supplementary Table S3. FoldX results for binding cavity amino acids (7Å within the N3 inhibitor). Catalytic dyad is marked **bold**.

Mutation	Energy [kcal/mol]	buried/exposed*
**H41L**	**-1.95**	**B**
H164L	-1.71	B
T169I	-1.49	E
T45S	-0.93	E
E47Q	-0.91	E
T24R	-0.84	E
S46P	-0.81	B
Q189L	-0.79	E
T26K	-0.78	E
**C145F**	**-0.77**	**B**
A191P	-0.58	E
H163Y	-0.55	B
E166Q	-0.43	E
L50R	-0.37	E
A193T	-0.36	E
H172L	-0.35	B
D187A	-0.35	E
V186F	-0.30	E
L141R	-0.28	B
N142Y	-0.22	B
N119Y	-0.08	E
S144A	-0.08	B
A173V	-0.02	B
T190I	0.05	E
Q192L	0.22	E
S147T	0.29	B
N28I	0.37	B
Y54F	0.40	B
C44S	0.41	B
M49L	0.42	B
V42L	0.74	B
Y118F	0.78	B
T25A	0.84	E
L27I	1.04	B
F140Y	1.09	B
F181Y	1.37	B
F185L	1.47	E
P52A	1.54	E
L167I	2.30	B
G143R	2.42	B
P39A	3.21	B
R40I	3.50	E
G146A	9.72	B

* based on the NetSurfP calculations

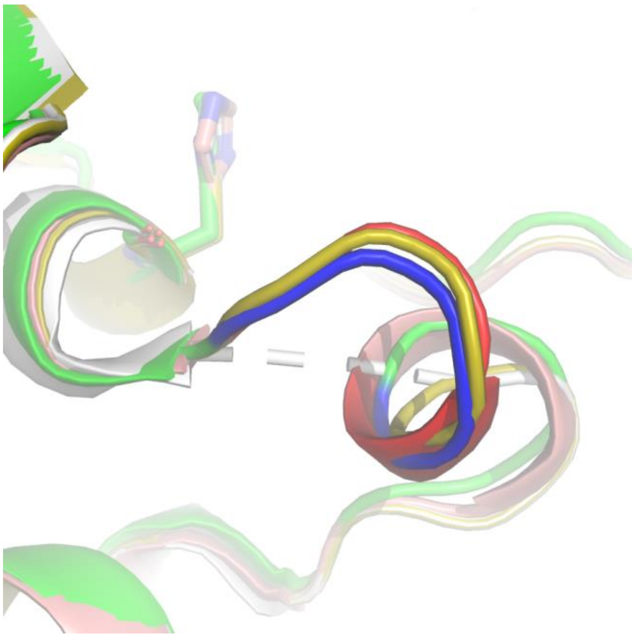

Supplementary Figure S8. Comparison of the rebuilded loop in 1q2w (gold), with loops from 6lu7 (blue) and 2h2z (red) structures. The white cartoon depicts original 1q2w pdb file. The H41 residue from active side dyad is shown in stick representation.

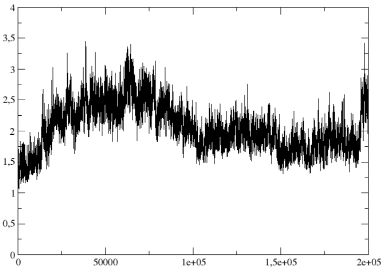

(a)

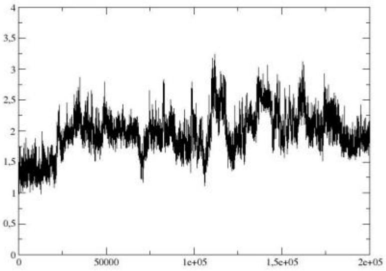

(b)

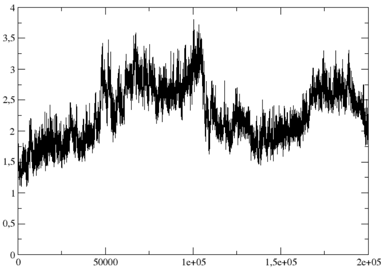

(c)

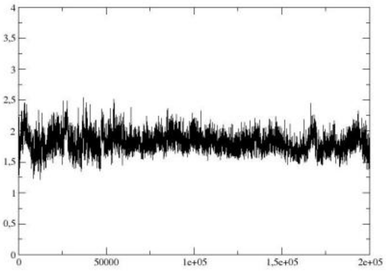

(d)

Supplementary Figure S9. RMSD calculated for 200 ns MD simulations of (a) SARS-CoV Mpro, (b) SARS-CoV Mpro N3, (c) SARS-CoV2 Mpro (d) SARS-CoV2 Mpro N3. The observed RMSD change comes from the III domain movement.

Supplementary Table S4. The number of added water molecules for classical MD simulations of SARS-CoV and SARS-CoV-2 Mpros.

Number of added molecules			
SARS-CoV-2 $Mpro^{N3}$	SARS-CoV-2 Mpro	SARS-CoV $Mpro^{N3}$	SARS-CoV Mpro
WAT: 22388	WAT: 19431	WAT: 23814	WAT: 22880

Supplementary Table S5. The final percentage concentration of particular cosolvents for both SARS-CoV-2 and SARS-CoV Mpros systems.

Cosolvent	Concentration [%]	Number of added molecules			
		SARS-CoV-2 $Mpro^{N3}$	SARS-CoV-2 Mpro	SARS-CoV $Mpro^{N3}$	SARS-CoV Mpro
ACN	4.5	ACN: 450<br>WAT: 19712	ACN: 450<br>WAT: 19924	ACN: 450<br>WAT: 19801	ACN: 450<br>WAT: 19858
BNZ	1.0	BNZ: 50<br>WAT: 19712	BNZ: 50<br>WAT: 19924	BNZ: 50<br>WAT: 19801	BNZ: 50<br>WAT :19858
DMSO	4.8	DMSO: 250<br>WAT: 19712	DMSO: 250<br>WAT: 19924	DMSO: 250<br>WAT: 19801	DMSO: 250<br>WAT: 19858
MEO	4.3	MEO: 550<br>WAT: 19712	MEO: 550<br>WAT: 19924	MEO: 550<br>WAT: 19801	MEO: 550<br>WAT: 19858
PHN	1.2	PHN: 50<br>WAT: 19712	PHN: 50<br>WAT: 19924	PHN: 50<br>WAT: 19801	PHN: 50<br>WAT: 19858
URE	4.4	URE: 300<br>WAT: 19712	URE: 300<br>WAT: 19924	URE: 300<br>WAT: 19801	URE: 300<br>WAT: 19858

Supplementary Table S6. Parameters for acetonitrile (ACN) molecules from the work of Nikitin and Lyubartsev [1].

Van der Waals parameters	R* (Å)	ε (kcal / mol)
YN	1.690	0.1331
YC	1.990	0.1341
CT	1.908	0.1094
HC	1.487	0.0157
**Bond parameters**	r_0 (Å)	K_b (kcal * mol-1 * Å-2)
CT-YC	1.458	400
YC-YN	1.157	600
CT-HC	1.090	340
**Angle parameters**	θ_0 (deg)	K_θ (kcal * mol-1 * rad-2)
CT-YC-YN	180	80
HC-CT-YC	110	35
HC-CT-HC	109.5	35
**Dihedral parameters**	Potential	Phase (deg)
X-CT-YC-X	0.0	60

[1] Nikitin, A.M.; Lyubartsev, A.P. New six-site acetonitrile model for simulations of liquid acetonitrile and its aqueous mixtures. *J. Comput. Chem.* **2007**, *28*, 2020–2026.

Supplementary Table S7. Parameters for benzene (BNZ) molecules obtained by Antechamber package.

Van der Waals parameters	R* (Å)	ε (kcal / mol)
cg	1.9080	0.2100
ch	1.9080	0.2100
**Bond parameters**	r_0 (Å)	K_b (kcal * mol-1 * Å-2)
cg-cg	1.377	494.2
cg-ch	1.191	949.5
ch-ch	1.377	494.2
**Angle parameters**	θ_0 (deg)	K_θ (kcal * mol-1 * rad-2)
cg-cg-ch	179.67	58.68
cg-ch-cg	179.67	58.68
cg-ch-ch	179.67	58.68
ch-cg-ch	179.67	58.68
**Dihedral parameters**	Potential	Phase (deg)
cg-cg-ch-ch	0.0	180

Supplementary Table S8. Parameters for dimethylsulfoxide (DMSO) molecules obtained by Antechamber package.

Van der Waals parameters	R* (Å)	ε (kcal / mol)
h1	1.3870	0.0157
s4	2.0000	0.2500
o	1.6612	0.2100
c3	1.9080	0.1094
Bond parameters	r_0 (Å)	K_b (kcal * mol-1 * Å-2)
c3-h1	1.093	335.9
o-s4	1.497	448.7
c3-s4	1.807	233.8
Angle parameters	θ_0 (deg)	K_θ (kcal * mol-1 * rad-2)
cg-cg-ch	179.67	58.68
cg-ch-cg	179.67	58.68
cg-ch-ch	179.67	58.68
ch-cg-ch	179.67	58.68
Dihedral parameters	Potential	Phase (deg)
cg-cg-ch-ch	0.0	180

Supplementary Table S9. Parameters for methanol (MEO) molecules obtained by Antechamber package.

Van der Waals parameters	R* (Å)	ε (kcal / mol)
h1	1.3870	0.0157
ho	0.0	0.0
oh	1.7210	0.2104
c3	1.9080	0.1094
Bond parameters	r0 (Å)	Kb (kcal * mol-1 * Å-2)
c3-h1	1.093	335.9
ho-oh	0.974	369.6
c3-oh	1.426	314.1
Angle parameters	θ0 (deg)	Kθ (kcal * mol-1 * rad-2)
h1-c3-h1	109.55	39.18
h1-c3-oh	109.88	50.97
c3-oh-ho	108.16	47.09
Dihedral parameters	Potential	Phase (deg)
h1-c3-oh-ho	0.5	0.0

Supplementary Table S10. Parameters for urea (URE) molecules based on the urea.frcmod file from Amber18 suite.

Angle parameters	θ0 (deg)	Kθ (kcal * mol-1 * rad-2)
N-C-N	112.6	71.3

Supplementary Table S11. Parameters for phenol (PHN) molecules obtained by Antechamber package.

Van der Waals parameters	R* (Å)	ε (kcal / mol)
oh	1.7210	0.2104
ca	1.9080	0.0860
ha	1.4500	0.0150
ho	0.0	0.0
**Bond parameters**	**r0 (Å)**	**Kb (kcal * mol-1 * Å-2)**
ca-oh	1.362	386.1
ho-oh	0.974	369.6
ca-ca	1.387	478.4
ca-ha	1.087	344.3
**Angle parameters**	**θ0 (deg)**	**Kθ (kcal * mol-1 * rad-2)**
ca-ca-oh	119.94	69.85
ca-oh-ho	109.47	48.85
ca-ca-ca	119.97	67.18
ca-ca-ha	120.01	48.46
**Dihedral parameters***	**Potential**	**Phase (deg)**
ca-ca-ca-oh	14.5	180.0
ha-ca-ca-oh	14.5	180.0
ca-ca-ca-ca	14.5	180.0
ca-ca-ca-ha	14.5	180.0
ca-ca-oh-ho	1.8	180.0
ha-ca-ca-ha	14.5	180.0

* two improper torsions: ca-ca-ca-oh and ca-ca-ca-ha with a penalty score=6.0
